# Defining the Glycosaminoglycan Interactions of Complement Factor H–Related Protein 5

**DOI:** 10.4049/jimmunol.2000072

**Published:** 2021-07-15

**Authors:** Frederick Gyapon-Quast, Elena Goicoechea de Jorge, Talat Malik, Nian Wu, Jin Yu, Wengang Chai, Ten Feizi, Yan Liu, Matthew C. Pickering

**Affiliations:** * Glycosciences Laboratory, Department of Metabolism, Digestion and Reproduction, Imperial College London, London, United Kingdom;; † Centre for Inflammatory Disease, Department of Immunology and Inflammation, Imperial College London, London, United Kingdom; and; ‡ Department of Immunology, Complutense University and Research Institute Hospital 12 de Octubre, Madrid, Spain

## Abstract

Complement activation is an important mediator of kidney injury in glomerulonephritis. Complement factor H (FH) and FH-related protein 5 (FHR-5) influence complement activation in C3 glomerulopathy and IgA nephropathy by differentially regulating glomerular complement. FH is a negative regulator of complement C3 activation. Conversely, FHR-5 in vitro promotes C3 activation either directly or by competing with FH for binding to complement C3b. The FH–C3b interaction is enhanced by surface glycosaminoglycans (GAGs) and the FH–GAG interaction is well-characterized. In contrast, the contributions of carbohydrates to the interaction of FHR-5 and C3b are unknown. Using plate-based and microarray technologies we demonstrate that FHR-5 interacts with sulfated GAGs and that this interaction is influenced by the pattern and degree of GAG sulfation. The FHR-5–GAG interaction that we identified has functional relevance as we could show that the ability of FHR-5 to prevent binding of FH to surface C3b is enhanced by surface kidney heparan sulfate. Our findings are important in understanding the molecular basis of the binding of FHR-5 to glomerular complement and the role of FHR-5 in complement-mediated glomerular disease.

## Key Points

- The pattern and degree of sulfation influenced FHR5–GAG interactions.
- Surface heparan sulphate enhanced the ability of FHR5 to limit FH binding to C3b.

## Introduction

The complement system is a complex group of proteins that contributes to host defense through effector pathways that result in surface opsonization, inflammatory cell recruitment, and membrane damage. A critical aspect of the system is the ability to regulate activation so that complement activation occurs appropriately, for example on the surface of a pathogen or damaged or dying cells, and not on healthy host tissue [reviewed in ref (1)]. Complement activation can occur following the binding of Abs to Ags (the classical pathway); following binding of pattern recognition receptors in the lectin pathway (e.g., the interaction between mannose-binding lectin and mannose-terminating glycans) and also spontaneously on surfaces through the activation of the alternative pathway (AP). Because of the ability of the AP to activate spontaneously and to contribute to amplification of surface complement generated through the lectin or classical pathways (AP amplification loop), regulation of this pathway is particularly important (2). Regulation of the AP is mediated by factor I (FI) and factor H (FH) [reviewed in (3)]). FI is an enzyme that, in the presence of a cofactor, cleaves the central complement activation protein C3b, preventing further activation. FH is an essential fluid-phase cofactor for the FI-mediated cleavage of C3b. FH also has a key role in determining whether surface C3b is inactivated by FI or can continue to amplify through the AP. FH consists of 20 domains, each of ∼60 amino acids termed short consensus repeat domains (SCR). FI cofactor activity is located within the first four SCR domains (SCR1-4, also termed complement regulatory domains). The C3b-binding domains are located within SCR1-4 and SCR19-20 (also termed complement recognition domains).

The interaction between FH and surface C3b depends not only on direct interaction with C3b through the recognition domains but critically on low-affinity interactions with glycan markers (“host polyanions”) namely, sulfated glycosaminoglycans (GAGs) and sialic acid–terminating glycans (4–9). The key role of sialic acid in influencing the ability of FH to regulate surface AP activation was first revealed in vitro: removal of sialic acid from sheep erythrocytes resulted in complement-mediated hemolysis due to loss of the protective interaction of FH with sialic acid on the erythrocytes surface (10). The FH–sialic acid recognition site is within SCR20 and there is an interaction with α2-3– (rather than α2-6– or α2-8–linked) *N-*acetylneuraminic acid (Neu5Ac) (4) and with *N*-glycolylneuraminic acid (Neu5Gc) (9). SCRs 6–8 and 19–20 contain GAG-binding sites. Binding to Bruch membrane in the eye is particularly dependent on interactions between SCR6-8 and heparan sulfate (HS), and to a lesser extent dermatan sulfate (DS) (7). In contrast, interaction within the kidney appears to be more dependent on SCR19-20–GAG interactions (7). The FH SCR19-20 domains are also important for interactions with endothelial cells (11). Mutations within this region (predominantly the SCR19-20 domains), result in impaired binding of FH to C3b, heparin and endothelial cells, and are associated with susceptibility to atypical hemolytic uremic syndrome, a thrombotic disorder of the kidney triggered by an inability to downregulate complement activation along the renal endothelium (12).

The regulation of C3b on surfaces by FH is also influenced by FH-related (FHR) proteins [reviewed in ref (13)]. There are five FHR proteins (FHR-1 to FHR-5); these, like FH, are composed of SCR domains of varying number and sequence similarity to those in FH. Importantly none of the FHR protein family contain complement regulatory domains comparable to those within FH SCR1-4 whereas FHR-1, -2, and -5 contain carboxyl terminal domains with sequence similarity to SCR19-20 domains of FH [reviewed in (14)]. Consequently, these proteins can compete with FH for binding to surface C3b (15, 16). But, unlike FH, they are unable to negatively regulate C3b amplification. In fact, FHR-1, FHR-4 (17), and FHR-5 (18) are each able to promote C3b activation in vitro. The biological relevance of the FHR proteins within the kidney is exemplified by the finding that FHR proteins influence surface complement activation even in the presence of intact FH proteins (19). The prototypical example of this was the observation that a heterozygous mutation in FHR-5 resulted in familial C3 glomerulopathy (20). FHR-5 is able to bind to C3b, and its cleavage products iC3b and C3d (15). It can also bind to laminins in basement membranes and to malondialdehyde-acetaldehyde epitopes on cell surfaces (21). In FHR-5 nephropathy the mutated FHR-5 protein is thought to promote C3 activation along surfaces within the kidney, by interfering with the ability of FH to negatively regulate glomerular complement by out-competing FH for binding to glomerular C3 (FH deregulation) (15). FHR-5 also influences the development of kidney injury in IgA nephropathy (22–24), a renal condition associated with abnormal accumulation of IgA and complement within the renal glomeruli. As FHR-5 is associated with both rare (C3 glomerulopathy) and common (IgA nephropathy) kidney diseases we wanted to elucidate the role of GAG ligands in the interaction between FHR-5 and renal complement. Using plate-based assays and GAG microarrays we demonstrate in this study the interactions of FHR-5 with GAG ligands and show that this interaction influences the binding of FH to surface C3b.

## Materials and Methods

### Proteins and Abs

Recombinant FHR-5 (rFHR-5) was transiently expressed in human embryonic kidney 293T cells as previously described (15). rFHR-5 was purified by affinity chromatography using the 2C6 Ab (mouse monoclonal anti-FHR1/2/5 Ab; Dr. Claire Harris, Newcastle University, Tyne, U.K.) cross-linked to an *N*-hydroxysuccinimide–activated Hitrap column (17071601; GE Healthcare). C3b and FH were sourced commercially (C3b-A114, FH-A137; Comptech). The homogeneity of the C3b was shown by SDS-PAGE (Supplemental Fig. 1). Desialylated FH (ΔSIA-FH) was prepared by incubating 250 µg FH with 0.3 U of agarose-conjugated *Clostridium perfringens* neuraminidase (N5254; Sigma-Aldrich) for 13 h at 37°C in 100 mM NaOAc, 2 mM CaCl_2_ buffer (pH 5.0). The Abs used in the current study include rabbit anti–full-length human FHR-5 (H00081494-D01; Abnova); mouse anti–human FHR-5 (H00081494-B01; Abnova); monoclonal mouse anti-human FH (OX24, MA1-70057; Pierce); and polyclonal goat antiserum to human FH (A312; Quidel), which was biotinylated using biotinamidohexanoic acid *N*-hydroxysuccinimide ester following protein G affinity purification. The molarity provided for FHR-5 is calculated for the dimer, which is its native form in circulation.

### GAGs

GAG polysaccharides sourced from Sigma-Aldrich include heparin (sodium salt from porcine intestinal mucosa, H3393), heparin-biotin sodium salt (B9806), HS (sodium salt from bovine kidney, H7640), DS (sodium salt from porcine intestinal mucosa, C3788), chondroitin sulfate A (CSA) (sodium salt from bovine trachea, C8529), chondroitin sulfate C (CSC) (sodium salt from shark cartilage, C4384), and hyaluronic acid (HA; sodium salt from bovine vitreous humor, H7630). HS (sodium salt from porcine intestinal mucosa, GAG-HS01), and 2-*O*-desulfated (DSH001/2), 6-*O*-desulfated (DSH002/6) and *N*-desulfated/reacetylated porcine intestinal mucosa heparin (DSH004/NAc) were from Iduron (Alderley Edge, England). GAG oligosaccharides were prepared by partial depolymerization of the polysaccharides using GAG specific lyases or nitrous acid and size-fractionated by gel filtration chromatography as previously described (25–27). The enzymatic digestions were monitored by absorption of UV 232 nm and terminated at 50% digestion. The chain lengths (the number of monosaccharide units or the degree of polymerization [DP]) of the major oligosaccharide components were determined by negative ion electrospray ionization mass spectrometry after conversion into their ammonium salts for the fractions with lower DPs (e.g., DP2 to DP6) (27) or deduced by gel filtration profiles of those with higher DPs (Supplemental Fig. 1).

### Plate-based GAG-binding assays

Heparin-binding plates (354676; BD Biosciences) were coated overnight with 50 µl GAG solution (40 µg/ml in 10 mM phosphate buffer, [pH 7.4], 150 mM NaCl [PBS]). This coating level was selected based on initial binding studies (not shown), which indicated that at 40 µg/ml, there was efficient coating of the positively charged (allyl amine) surface not only with the highly sulfated GAG, heparin, but also the nonsulfated hyaluronan and the low sulfated CSA. The coating with hyaluronan was detected using the carbohydrate-binding module of *Streptococcus pneumoniae* hyaluronate lyase (28) and with CSA using mAb CS-56 (29). Wells were washed twice with 100 µl wash buffer (10 mM HEPES, 150 mM NaCl [pH 7.3]) to remove unbound GAGs and blocked with Carbo-Free blocking reagent (SP-5040; Vector Laboratories) for 2 h at room temperature. After washing the wells four times with 100 µl of wash buffer, serial dilutions of 50 µl of FH or rFHR-5 were added and incubated for 2 h. Surface-bound FH was detected using biotinylated goat anti-human FH (1 µg/ml; in-house preparation from goat antiserum to FH; A312; Quidel) and streptavidin-HRP conjugate (1:200 dilution, DY998; R&D Systems). Surface-bound rFHR-5 was detected using mouse anti–FHR-5 (1 µg/ml), HRP-conjugated rabbit anti-mouse IgG (1:1000 dilution, P0260; DAKO/Agilent). The same chromogenic substrate 3,3′,5,5′-tetramethylbenzidine (555214; BD Biosciences,) was used throughout. As protein and Ab diluent 5% w/v BSA (A7030; Sigma-Aldrich) was used in acetate buffer (50 mM NaOAC, 100mM NaCl, [pH 7.3]). The colorimetric reaction was terminated by adding 20 µl of 2 N sulfuric acid. The OD was measured at 450 nm using a spectrophotometer (Multiskan Ascent; Thermo Fisher Scientific). After each incubation with protein/Ab the wells were washed four times with 100 µl wash buffer. Nonspecific binding signals of FH or rFHR-5 to the positively charged polymer-surface (wells not coated with GAG) as well as Ab alone signals (GAG-coated wells incubated with detection Abs, in absence of FH or rFHR-5) were subtracted from the raw signals.

### GAG oligosaccharide microarray

Microarray analyses were carried out using the neoglycolipid (NGL)-based microarray system (30). Details of the glycan probe library, the generation of the microarrays, imaging and data analysis are in a glycan microarray document (Supplemental Table I) in accordance with guidelines for reporting glycan microarray-based data: Minimum Information Required for A Glycomics Experiment (31). Microarray analyses of FH and rFHR-5 were performed at ambient temperature. In brief, the arrayed slides were blocked for 60 min with 3% BSA (w/v) in PBS. The proteins were diluted in a 1% BSA-PBS (w/v) solution at 50, 10, and 5 µg/ml (Fig. 2) and overlaid onto the preblocked microarray surface for 90 min. Binding of FH was detected using goat anti-human FH serum (1:200 dilution; Quidel) and rFHR-5 was detected using polyclonal mouse anti–FHR-5 (5 µg/ml; Abnova) followed by biotin-conjugated rabbit anti-goat IgG (1:200 dilution, B7014; Sigma-Aldrich) and biotin-conjugated goat anti-mouse IgG (1:200 dilution, B7264; Sigma-Aldrich). For final signal detection, the arrays were incubated with Alexa 647–conjugated streptavidin (1 µg/ml; Molecular Probes) for 30 min. The sequences of the 63 GAG NGL probes included in the focused GAG array set (GAG oligosaccharide array set 4) are in Supplemental Table II. Binding signals were glycan dose related. The results at 5 fmol NGL probe per spot are presented as histogram charts (Fig. 2) and Supplemental Table III, which include the fluorescence intensities and error bars (half of the difference of signal intensities of duplicate spots of each glycan probe). Additional analyses of rFHR-5 binding were carried out at a lower concentration range (10, 5, 1, and 0.2 µg/ml) (Fig. 3) using a more recent GAG array (in-house designation “GAG Oligosaccharide Array Set 11”) that included two HS and four heparin oligosaccharide probes.

### Inhibition assays of heparin binding by fluid-phase GAGs

Heparin-binding plates were coated overnight with 100 µl of 20 µg/ml heparin in PBS solution. Wells were washed twice with 100 µl wash buffer to remove unbound heparin and blocked with 100 µl of Carbo-Free blocking reagent (1:10 dilution) for 2 h at room temperature. rFHR-5 was diluted in 5% BSA-binding buffer (5% BSA w/v, 50 mM NaOAC, 100 mM NaCl [pH 7.3]) to 1.5 µg/ml for incubation with GAG polysaccharides and diluted to 0.5 µg/ml for incubation with heparin oligosaccharides (32). rFHR-5 was preincubated with GAG polysaccharides (160 µg/ml) or heparin oligosaccharides (160 µg/ml) for 1 h at 4°C. For assessment of the influence of ionic strength on the rFHR-5/FH heparin interaction in the fluid-phase, rFHR-5 (1.5 µg/ml) and FH (0.5 μg/ml) solutions were preincubated for 1 h with 160 µg/ml heparin solutions, which were prepared in buffers (5% BSA w/v, 20 mM Tris) with different molarities of NaCl (20, 40, 50, 60, 80, 100, 150 mM). Following this, 50 µl of the protein-GAG mixtures were transferred onto the heparin-coated plates. After 2 h of incubation at room temperature, the wells were washed four times with 100 µl wash buffer. Detection of the surface-bound FH and rFHR-5 was performed as described above, using anti-human FH or anti–FHR-5 Abs followed by secondary Abs and color development. The signals were corrected against Ab blank values and nonspecific protein binding to the plate surface as described above. The OD was displayed as percentage of rFHR-5/FH binding using as 100% the OD values of FHR-5 or FH in the absence of soluble heparin.

### Analyses of FH binding to surface-bound C3b with or without HS in the presence of rFHR-5

Heparin-binding plates were coated overnight at 4°C with 25 µg/ml human C3b alone or in combination with either 40 µg/ml kidney HS and 40 µg/ml HA, respectively. Following a wash step to remove any unbound C3b or GAG, the plates were blocked with 100 µl of 1× Carbo-Free for 2 h at room temperature. Increasing concentrations of rFHR-5 (7, 14, 28, 55, 111, and 221 nM; corresponding to a concentration range of 1 to 30 µg/ml of dimeric rFHR-5) were added to fixed concentration of FH 16 nM (2.5 µg/ml) in 5% BSA-binding buffer (5% BSA w/v, 100 mM NaCl, 50 mM NaOAC [pH 7.3]) and 50 µl thereof incubated in the wells for 2 h at room temperature. Surface-bound FH was detected using a monoclonal mouse IgG anti-FH (2.5 µg/ml, Invitrogen/Thermo Fisher Scientific, MA1-70057) followed by HRP-conjugated polyclonal rabbit IgG anti-mouse IgG (1:1000 dilution, P0260; DAKO/Agilent). FH binding in the absence of rFHR-5 was denoted 100%.

## Results

### rFHR-5 and FH bind to sulfated GAG polysaccharides and the interaction is influenced by the degree and the pattern of sulfation

**FIGURE 1.**
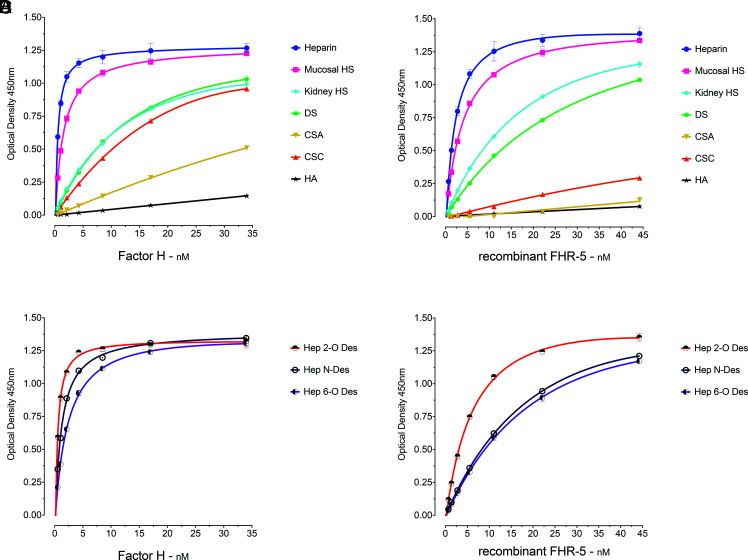
Binding of FH and rFHR-5 to surface-immobilized GAGs. (**A**) FH and (**B**) rFHR-5 showed dose-dependent binding correlating with the degree of GAG sulfation and lack of binding to the nonsulfated GAG HA. (**C**) FH and (**D**) rFHR-5 binding to surface-immobilized, selectively desulfated GAGs showing reduction of binding after desulfation. The data are representative of more than 10 independently performed experiments using the GAGs and representative of two independently performed experiments using the selectively desulfated GAGs. Data points represent mean of two replicates with SD. Hep 2-*O*-deS, heparin desulfated at the 2-O position of the iduronic acid; Hep 6-*O*-deS, heparin desulfated at the 6-O position of the iduronic acid; Hep N-deS, heparin desulfated at the N-position of the glucosamine followed by N-reacetylation.

To examine the GAG-binding properties of FH and FHR-5, we first analyzed the binding of serum-derived FH and rFHR-5 to GAG polysaccharides immobilized in heparin-binding plates (Fig. 1). The polysaccharides included heparin, HS derived from porcine intestinal mucosa and bovine kidney, DS, CSA, and CSC, and HA. The two proteins were added to the wells at different concentrations and the binding signals analyzed. The strongest binding by both proteins was to the most highly sulfated GAG, heparin, and no interaction was evident with the nonsulfated polysaccharide HA (Fig. 1). The binding to porcine intestinal mucosal HS was slightly less intense than to heparin. Lower binding intensities were observed between FH and bovine kidney HS, DS, and CSC, followed by CSA (Fig. 1A). A broadly similar binding profile was seen for rFHR-5, including relatively strong binding to heparin, HS, and DS. Both proteins interacted less strongly with HS from kidney compared with HS from porcine intestinal mucosa. Although FH bound relatively strongly to CSC, rFHR-5 showed only weak binding to CSC and little binding to CSA (Fig. 1B). The results correlate with the degree of sulfation which progressively decreases from heparin (27) to intestinal mucosal (33) and kidney HS (34).

The majority of heparin disaccharide units contain three sulfate groups (with 2-*O*-sulfate at iduronic acid and 6-*O*-sulfate and 2-*N*-sulfate at glucosamine residues). To characterize the influence of pattern of sulfation on FH and rFHR-5 binding, we compared the interactions of the proteins with heparin and selectively modified forms of heparin (Fig. 1C, 1D). We used heparins that were chemically 2-*O*-desulpahted, 6-*O*-desulfated, or *N*-desulfated and re–*N*-acetylated. Both FH (Fig. 1C) and rFHR-5 (Fig. 1D) showed a reduction in binding strength to 6-*O*-desulfated heparin. rFHR-5 showed reduction in binding to *N*-desulfated heparin to a similar extent as 6-*O*-desulfated, whereas *N*-desulfation had less impact on FH binding. The rFHR-5 showed reduced binding to 2-*O*-desulfated heparin in comparison with its binding to heparin, although to a lesser extent compared with the 6-*O*-desulfated or *N*-desulfated heparin. Binding of FH to 2-*O*-desulfated and heparin was almost identical. Clearly, the pattern of sulfation has an effect on the binding intensities of the two proteins. In summary, the 6-*O*-sulfation has a similar contribution to the binding strengths of the two proteins whereas the influences of *N*-sulfation and 2-*O*-sulfation are greater for rFHR-5 than for FH.

### rFHR-5 and FH share similar GAG oligosaccharide-binding profiles but of differing strength

**FIGURE 2.**
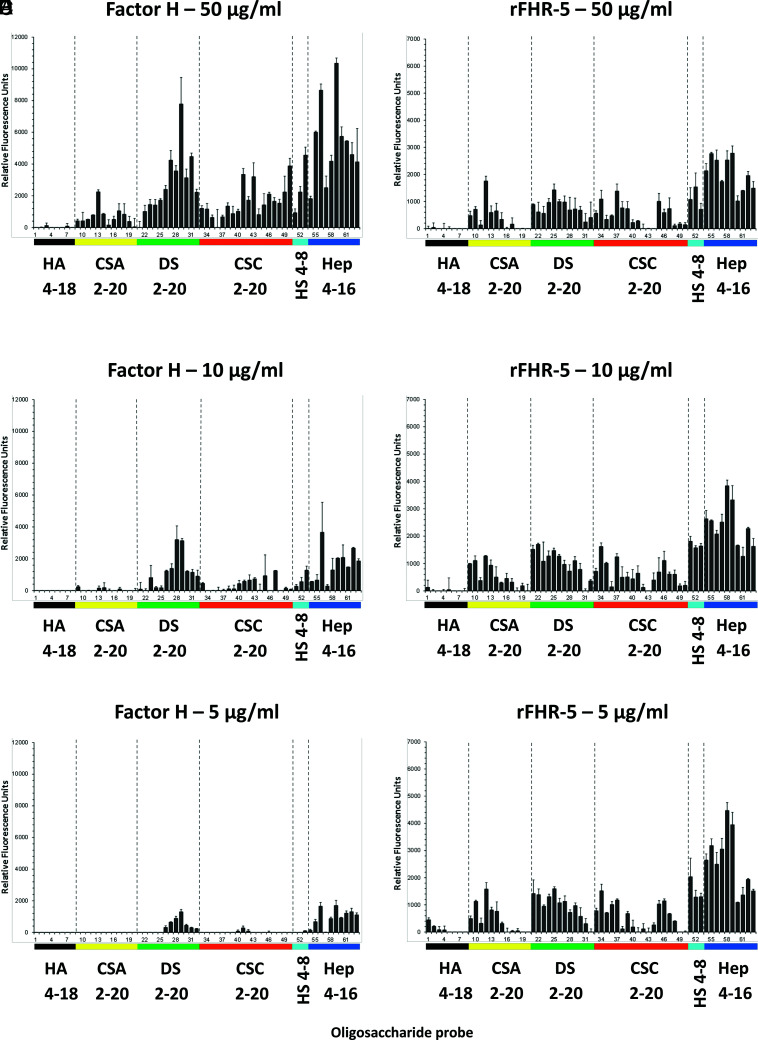
Binding of FH and rFHR-5 to GAG oligosaccharide microarrays. FH (**A**–**C**) or rFHR-5 (**D**–**F**) were applied to GAG oligosaccharide microarrays at concentrations of 50, 10, and 5 µg of protein per milliliter. Results with increasing oligosaccharide chain lengths are shown from left to right. Sequences of the GAG NGL probes (numbered 1–63 and listed in Table I) are in Supplemental Table II. Binding signals (fluorescence intensities) represent means of duplicates at 5 fmol per spot of each oligosaccharide probe. Error bars represent half of the difference between two values. Binding data shown are representative of at least two independent experiments. Hep, heparin.

**FIGURE 3.**
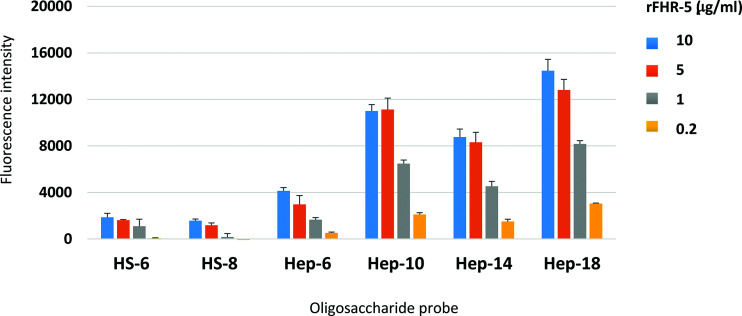
Protein concentration-dependent binding of rFHR-5 to HS and heparin oligosaccharide microarrays. FHR-5 was overlaid at 10, 5, 1, and 0.2 µg of protein per milliliter onto microarrays that contain HS and heparin NGL probes with selected saccharide chain lengths as indicated. Binding signals (fluorescence intensities) represent mean of quadruplicate spots at 5 fmol/spot and error bars represent SD. Binding data shown are representative of at least two independent experiments.

The GAG-binding properties of FH and FHR-5 were further investigated using a GAG oligosaccharide microarray which contains NGLs of 63 size-defined oligosaccharide fractions derived from HA, CSA, DS, CSC, HS, and heparin (Fig. 2, Table I). The GAG NGL arrays were overlaid with FH and rFHR-5 each at three concentrations (50, 10, and 5 µg/ml). The two protein preparations bound exclusively to the sulfated GAG oligosaccharide probes and not to the nonsulfated HA probes. Both proteins showed preferential binding to oligosaccharide probes of heparin followed by DS derived probes, in accord with observations with plate assays (Fig. 1). The binding signals with FH were dependent on protein concentrations at the levels tested (Fig. 2A–C) but not with rFHR-5 (Fig. 2D–F), indicating a higher avidity of binding for the latter. This was confirmed using an array encompassing 6 and 8 mers of HS and 6, 10, 14, and 18 mers of heparin, where rFHR-5 showed protein concentration-dependent binding to these HS and heparin oligosaccharide probes when the protein was overlaid at concentrations lower than 5 µg/ml (Fig. 3).

**Table I.** List of GAG NGLs in the GAG oligosaccharide microarrays

Position	Probe Name	Position	Probe Name	Position	Probe Name
1	HA-S4	22	DS-5	43	CSC-18
2	HA-S6	23	DS-2	44	CSC-20
3	HA-S8	24	DS-4	45	CSC-S4
4	HA-S10	25	DS-6	46	CSC-S6
5	HA-S12	26	DS-8	47	CSC-S8
6	HA-S14	27	DS-10	48	CSC-S10
7	HA-S16	28	DS-12	49	CSC-S12
8	HA-S18	29	DS-14	50	CSC-S14
9	CSA-3	30	DS-16	51	HS-S4-AO
10	CSA-5	31	DS-18	52	HS-S6-AO
11	CSA-2	32	DS-20	53	HS-S8-AO
12	CSA-4	33	CSC-3	54	Hep-S4-AO
13	CSA-6	34	CSC-5	55	Hep-S6-AO
14	CSA-8	35	CSC-2	56	Hep-S8-AO
15	CSA-10	36	CSC-4	57	Hep-4-AO
16	CSA-12	37	CSC-6	58	Hep-6-AO
17	CSA-14	38	CSC-8	59	Hep-8-AO
18	CSA-16	39	CSC-10	60	Hep-10-AO
19	CSA-18	40	CSC-12	61	Hep-12-AO
20	CSA-20	41	CSC-14	62	Hep-14-AO
21	DS-3	42	CSC-16	63	Hep-16-AO

### The binding of rFHR-5 to surface-bound heparin is inhibited by fluid-phase preincubation with sulfated GAGs

**FIGURE 4.**
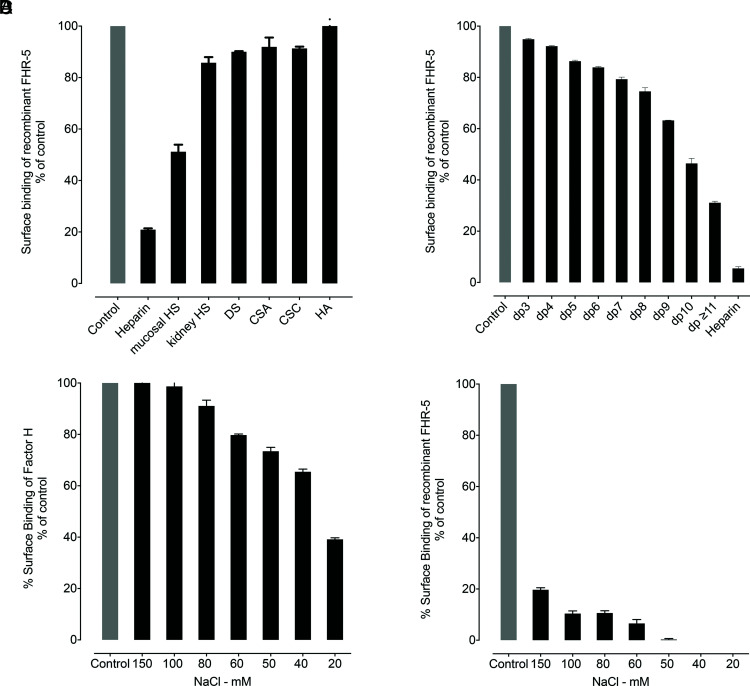
The binding of rFHR-5 to surface heparin is inhibited by fluid-phase preincubation with sulfated GAGs. (**A**) Binding of rFHR-5 (1.5 µg/ml) to surface heparin following preincubation with GAG polysaccharides (160µg/ml). (**B**) Binding of rFHR-5 (0.5 µg/ml) to surface heparin following preincubation with heparin oligosaccharides (160 µg/ml). Control represents surface binding of rFHR-5 in the absence of fluid-phase GAGs. The effect of ionic strength on the fluid-phase inhibition by heparin (160 µg/ml) of the surface binding of (**C**) FH (0.5 µg/ml), and (**D**) rFHR-5 (1.5 µg/ml) to immobilized heparin. The data are representative of two independently performed experiments. Data points represent mean of two replicates with SD.

To investigate further the ability of FH and rFHR-5 to interact with GAG polysaccharides in the fluid-phase, we incubated the proteins with different GAG polysaccharides and then examined how this affected the interaction of the two proteins with surface-bound heparin. The binding of rFHR-5 to immobilized heparin was inhibited by preincubation with heparin polysaccharide (Fig. 4A). No inhibition was observed following preincubation with HA. The degree of inhibition seen following preincubation with other GAG polysaccharides in the fluid-phase correlated with the degree of GAG sulfation: The greatest inhibition was with intestinal mucosal HS. To determine the minimal GAG oligosaccharide chain length required for the interaction of rFHR-5 and soluble GAG polysaccharides, we preincubated rFHR-5 with heparin oligosaccharides of defined chain lengths (2 to 20 mers). The degree of inhibition of rFHR-5 to surface heparin increased as the chain length of the soluble heparin increased (Fig. 4B). In contrast to rFHR-5, fluid-phase preincubation of FH with heparin polysaccharide at physiological ionic strength did not affect the strength of FH binding to surface heparin. Only when the ionic strength was reduced to below 100mM NaCl were we able to detect inhibition of binding to surface heparin (Fig. 4C, 4D).

### Surface-bound sulfated GAG influences the relative binding strengths of FH and rFHR-5 to C3b

**FIGURE 5.**
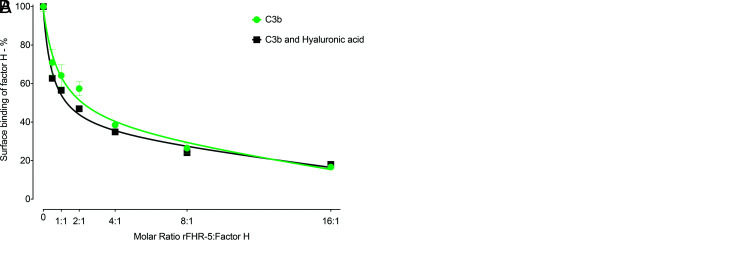
Influence of surface-coated HS on the relative binding of FH and rFHR-5 to surface C3b. (**A**) FH binding to either surface C3b (green) or surface C3b together with hyaluronic acid (black) in the presence of increasing concentrations of rFHR-5. The data are from one experiment. Data points represent mean of two replicates with SD. (**B**) FH binding to either surface C3b (green) or surface C3b together with kidney HS (red) in the presence of increasing concentrations of rFHR-5. The data are representative of two independently performed experiments. Data points represent mean of two replicates with SD.

FHR-5 can act as a competitive antagonist of FH, a process termed FH de-regulation (15). To explore the contribution of the protein interactions with polysaccharides to this process, we examined the ability of rFHR-5 to inhibit the interaction of FH with surfaces coated with either C3b alone, with C3b and kidney HS, or with C3b and HA. As previously shown (15), there was a dose-dependent inhibition of the binding of FH to C3b as the concentration of rFHR-5 was increased (Fig. 5). This dose-dependent inhibition of FH surface binding was comparable when surfaces coated with C3b alone and with both C3b and HA (Fig. 5A). However, inhibition of FH surface binding by rFHR-5 was enhanced on surfaces coated with C3b and kidney HS compared with surfaces coated with C3b alone (Fig. 5B). At a 1:1 molar ratio of rFHR-5 and FH (Fig. 5B) surface FH binding to C3b alone was reduced to ∼ 80% but fell to ∼ 50% when surfaces were coated with C3b and kidney HS. These data indicate that the immobilized sulfated polysaccharide can alter binding of FH and FHR-5 to surface C3b.

## Discussion

We have explored the interaction between FHR-5 and GAG ligands using both a plate-based assay and a carbohydrate microarray. Using the plate-based assay the binding profiles of FH and rFHR-5 were broadly similar and correlated with the degree of sulfation of the GAGs examined, with strongest binding to heparin and no binding to HA. However, there were some differences in that, whereas FH bound strongly to CSC the rFHR-5 did not. The significance of this is unclear. The predominant GAG in human glomerular and tubular basement membranes is HS, constituting at least 75% of total GAGs present (35). However ∼ 20% is CS (35) and differences in the interaction of FH and FHR-5 with renal basement membrane CS may be relevant during complement activation on these surfaces. Based on our data using selectively desulfated heparins, we deduce that the pattern, as well as the degree of GAG sulfation, influences the interaction between rFHR-5 and heparin. This has previously been shown for the FH interactions (7). Using FH, the SCR19-20 domains and immobilized heparins, the interaction progressively diminished following *N*-desulfation-*N*-reacetylation; 2-*O*-desulfation; 6-*O*-desulfation; and 2,6-*O*-desulfation (7). Using a similar approach, we corroborated reduction in FH binding to heparin after desulfation, and showed that this also occurs for rFHR-5. In our studies the contributions of *N*-sulfation and 2-*O*-sulfation to heparin binding were greater for rFHR-5 than for FH.

To further characterize the GAG interactions of FHR-5 we studied the interaction of rFHR-5 with 63 NGLs derived from HA, CSA, DS, CSC, HS, and heparin (Supplemental Table II). The NGLs were derived from size-defined GAG chains and we demonstrated that rFHR-5 bound only to sulfated GAG oligosaccharide probes. Interestingly, although both FH and FHR-5 showed greater binding to heparin and DS probes, a protein dose–dependent interaction was evident only at a lower protein concentration range for FHR-5 than FH. This indicates that the interaction between FHR-5 and these NGLs is stronger, and this may derive from the fact that FHR-5 exists as an obligate “head-to-tail” dimer mediated by the first two N-terminal SCR domains (15). FHR-1, FHR-2, and FHR-5 share high sequence similarity within these first two domains and all circulate as homodimers. Although heterodimerization is possible, studies have shown that the only heterodimer to be identified physiologically is the FHR-1–FHR-2 heterodimer (36). So, our use of rFHR-5 homodimers corresponds to the FHR-5 that is circulates in vivo.

We were able to inhibit the binding of rFHR-5 to immobilized heparin by preincubation of rFHR-5 with heparin. This inhibition was dependent on sulfation as 1) no inhibition was seen if rFHR-5 was preincubated with HA; 2) the degree of inhibition with other GAGs correlated with the degree of sulfation; and 3) inhibition increased as the chain length of the heparin oligosaccharide increased. These data suggest that FHR-5 may interact with GAGs in the fluid-phase in vivo. Interestingly, preincubation of FH with heparin reduced the subsequent binding to surface heparin only when the ionic strength was below physiological sodium levels. The inhibition studies with FH were not pursued further.

FHR-5 has been shown to be present in association with complement C3 within glomeruli in a variety of glomerular conditions (37). And mutations in FHR-5 are associated with C3 glomerulopathy (19, 20). Using surface plasmon resonance it has been shown that FHR-5 binds to the C3 activation fragments: C3b, iC3b, and C3dg (15). FH binds to C3b and the association between abnormal FHR-5 proteins and C3 glomerulopathy has been proposed to be due to an ability of FHR-5 to out-compete FH for interaction with C3b. This has been demonstrated in vitro using hemolytic assays (15). However, the contribution of GAG ligands to the binding of FHR-5 to surface C3b had not been explored. Our data show that surface-bound sulfated GAG (kidney HS) influences the interactions of rFHR-5 and FH with immobilized C3b. Whereas the binding of FH to surface C3b could be reduced by rFHR-5 independently of the presence of surface GAG, this reduction was enhanced when surface GAG was present. These data indicate that GAGs in the kidney can influence to the interaction of FHR-5 with renal C3b.

The biological roles of FHR-5 and of the other FHR proteins are still poorly understood. FHR-5 is present within glomeruli in C3 glomerulopathy (38), IgA nephropathy (23), and other glomerular conditions associated with complement activation (37). Understanding the molecular basis of this interaction will be key to designing approaches to prevent it. The FHR-5–heparin interaction was located within FHR-5 SCR5-7 (21, 39). The subunit domains of the FHR-5 protein that mediate other GAG interactions remain to be defined. The influence of the FHR-5–GAG interaction on other binding partners of FHR-5 also remains unclear; these ligands include properdin (40) and laminins (21) and pentraxin-3 (18). Specifically, FHR-5 binds to laminin-521 and laminin-211 and this may be relevant to FHR-5 binding within the kidney as these laminins are components of the glomerular basement membrane and mesangial matrix, respectively.

In sum, we have studied the interactions of FHR-5 with GAG ligands using both plate-based assays and a microarray platform and shown that the interaction of rFHR-5 with kidney HS reduces the ability of FH to interact with surface C3b. Understanding the molecular basis of the FHR-5–GAG interactions will be important in elucidating the relationship between glomerular complement activation and FHR-5.

## Supplementary Material

Data Supplement
