# Supplementary material for: Defining the glycosaminoglycan interactions of complement factor H-related protein 5
Source: J Immunol. Author manuscript; Available in PMC 2021 Sep 24. (PMC8313009; doi:10.4049/jimmunol.2000072)
Supplement: Supplemental Figure 1 [file EMS124726-supplement-Supplemental_Figure_1.pdf]

A

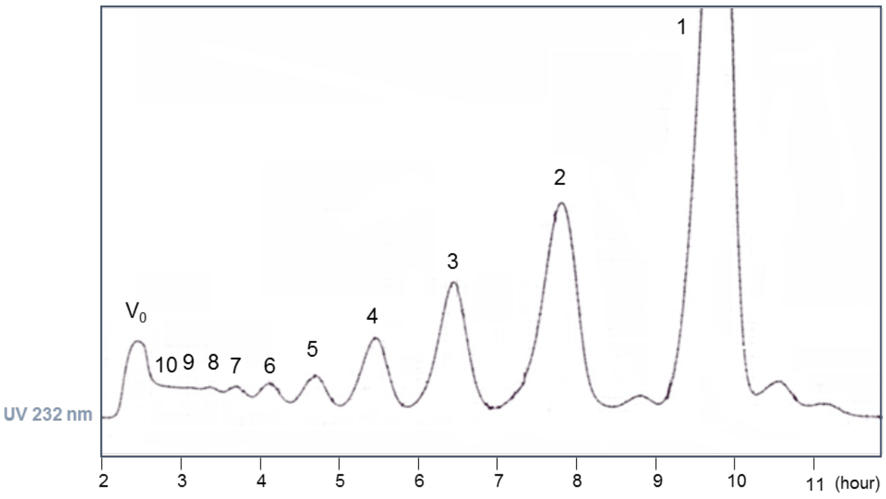

	Detected major ions	Deduced compositions	Assignment
F1	m/z 458.0605 (z=1)<br>m/z 561.0070 (z=1)	DUA1.GalNAc1.S1<br>DUA1.GalNAc1.S2.Na	DP2
F2	m/z 458.0605 (z=2)<br>m/z 837.1719 (z=1)	DUA1.GlcA1.GalNAc2.S2<br>DUA1.GlcA1.GalNAc2.S1	DP4
F3	m/z 709.5765 (z=2)	DUA1.GlcA2.GalNAc3.S3.Na2	DP6

B

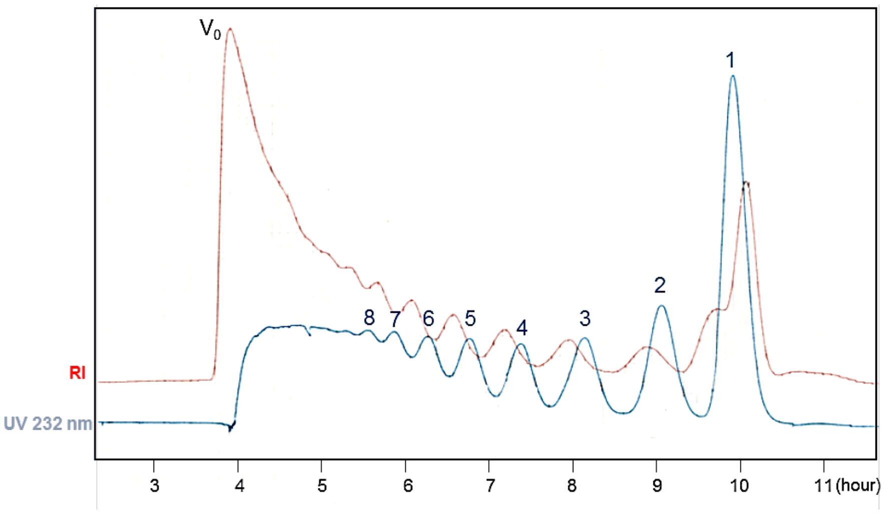

	Detected major ions	Deduced compositions	Assignment
F1	m/z 286.9740 (z=3)	DUA1.HexN1.S3	DP2
F2	m/z 213.7894 (z=5)<br>m/z 229.7807 (z=5)	DUA1.HexA1.HexN2.S5<br>DUA1.HexA1.HexN2.S6	DP4

Supplemental Figure 1. Fractionation and assignment of GAG oligosaccharide fractions of CSC (A) and heparin (B). In each panel the Bio-Gel P6 chromatograph of the oligosaccharide fractions derived from partial enzyme digestion is shown followed by the table showing assignment of selected oligosaccharide fractions by negative-ion ESI-MS. DUA, 4,5-unsaturated uronic acid; GlcA, glucuronic acid; GalNAc, N-Acetylgalactosamine; HexA, hexuronic acid; HexN, Hexamine; S: sulfate. The numbers in the deduced compositions refer to monosaccharides, sulfates and sodium (Na) per molecules of oligosaccharide. DP, degree-of-polymerisation.
